# The individual’s signature of telomere length distribution

**DOI:** 10.1038/s41598-018-36756-8

**Published:** 2019-01-24

**Authors:** Simon Toupance, Denis Villemonais, Daphné Germain, Anne Gegout-Petit, Eliane Albuisson, Athanase Benetos

**Affiliations:** 1 Université de Lorraine, Inserm, DCAC, F-54000 Nancy, France; 2 Université de Lorraine, CHRU-Nancy, Pôle “Maladies du Vieillissement, Gérontologie et Soins Palliatifs”, F-54000 Nancy, France; 3 Nancyclotep-GIE, F-54000 Nancy, France; 4 Université de Lorraine, Ecole des Mines, F-54000 Nancy, France; 5 Université de Lorraine, CNRS, Inria, IECL, F-54000 Nancy, France; 6 Université de Lorraine, CHRU de Nancy, BIOBASE, Pôle S2R, Nancy, F-54000 France; 7 Université de Lorraine, InSciDenSe, F-54000 Nancy, France

## Abstract

Mean telomere length in human leukocyte DNA samples reflects the different lengths of telomeres at the ends of the 23 chromosomes and in an admixture of cells. However, only rudimentary information is available regarding the distribution of telomere lengths in all chromosomes and the different cell types in leukocyte samples. Understanding the configuration of leukocyte telomere length distribution (LTLD) could be helpful in capturing intrinsic elements that are not provided by the mean leukocyte telomere length (mLTL). The objective of this study was to analyse LTLD and its temporal variation in adults. Leukocyte samples were donated on two occasions (8 years apart) by 72 participants in the ADELAHYDE study. Telomere length was measured by Southern blotting of the terminal restriction fragments. Individuals with comparable mLTLs displayed different shapes of LTLDs. Inter-individual variation in LTLD shape was much larger than intra-individual variation in LTLD shape between baseline and follow-up leukocyte samples. These results show an important individual stability of LTLD shape over time indicating that each individual has a characteristic LTLD signature.

## Introduction

Telomeres are non-coding nucleoprotein structures located at the end of chromosomes. They protect loss of vital DNA sequences, block end-to-end fusions and facilitate distinguishing chromosome ends from DNA damage^1^.

With age, telomere length (TL) displays a progressive shortening in replicating somatic cells due to the “end replication problem”^2^. Ultimately, these cells acquire critically short and dysfunctional telomeres that lead to growth arrest known as replicative senescence^3^. A body of work has focused on examining the potential role of TL dynamics (TL and its age-related shortening) in aging and its related diseases. In adults, short leukocyte TL (LTL), a proxy to other somatic cells^4^, is associated with a higher risk of cardiovascular^5,6^, neurodegenerative^7,8^ and metabolic diseases^6,9^, as well as with untimely death^10^.

Several methods have been developed to measure TL, including 1) Southern blot (SB) analysis of the terminal restriction fragments (TRF)^11^; 2) quantitative PCR (qPCR) whose output is expressed as the ratio of telomere product relative to a single copy gene product^12^; 3) fluorescent *in situ* hybridization (FISH) techniques, including quantitative FISH (qFISH) based on microscopy and flow FISH using flow cytometry^13^; 4) single telomere length analysis (STELA)^14^ and telomere shortest length assay (TeSLA)^15^, measurements at the single telomere level based on a combination of PCR and Southern blot. There is ongoing debate as to which method of TL measurement, principally SB vs. qPCR^16–18^, is optimal in epidemiological settings. The advantages of the qPCR method are its high throughput and low cost. SB, considered the “gold standard”^19^, yields both mean TL (mTL) values and generates the distribution of TLs in the DNA sample^20^. However, the vast majority of studies using SB measurements only report mTL and not the parameters of TL distribution. Generating data on TL distribution can provide valuable information, since the shortest telomeres, rather than mTL, may represent the main determinants of cell fate^14,21–23^. Accordingly, the aim of this study was to analyse the distribution of telomere length in leukocytes and its reconfiguration over the course of 8 years in 72 subjects from the ADELAHYDE (Analyse des DEterminants génétiques et environnementaux de la Leucoaraïose dans une population à sujets Agés HYpertendus présentant des troubles cognitifs DEbutants) study (Table 1).

**Table 1:** Characteristics of the participants of the ADELAHYDE cohort.

	All	Men	Women
Number of participants	72	36	36
Age at baseline (years)	67.7 ± 5.4	67.5 ± 5.4	68.0 ± 5.5
Age at follow-up (years)	75.9 ± 6.0	75.8 ± 6.1	76.0 ± 6.0
Follow-up duration (years)	8.2 ± 1.2	8.4 ± 1.4	8.0 ± 1.0
mLTL at baseline (kb)	6.39 ± 0.57	6.29 ± 0.53	6.49 ± 0.60
mLTL at follow-up (kb)	6.16 ± 0.57	6.06 ± 0.54	6.26 ± 0.58
mLTL attrition (bp/year)	27.3 ± 25.6	27.4 ± 23.6	27.2 ± 27.8

mLTL, mean leukocyte telomere length.

Values are mean ± SD or %.

We hypothesized that the leukocyte telomere length distribution (LTLD) evolved over the 8-year follow-up period and that changes in shape would be noticeable. This hypothesis was based on the following two statements. First, when TL is studied with the TRF method, the results reflect the telomere length at both ends of the 23 pairs of chromosomes (i.e. 92 telomeres for one cell). Attrition can cause a single short telomere to reach the limit that will trigger cell senescence. In this case, this short telomere will disappear as well as the 91 others of this same cell. The impact of such successive losses along years on the shape of the distribution can be assumed to be noticeable. Secondly TRF measurements involve a mixture of cells and the LTLD reflects also the variability of TL among these different cells. Indeed, TL in leukocyte subtypes can differ^24^ and the “composition” in leukocyte subtypes can vary between baseline and follow-up, depending on the inflammatory status of the patient, leading to noticeable LTLD evolutions.

## Results

### LTL parameters

Mean LTL decreased between baseline and follow-up (Table 1; p < 0.0001). There was a trend for longer mLTL in women than in men both at baseline (p = 0.15) and follow-up (p = 0.13). The average rate of mLTL attrition was 27.3 base pairs (bp) per year.

### mLTL tracking and ranking

**Figure 1:**
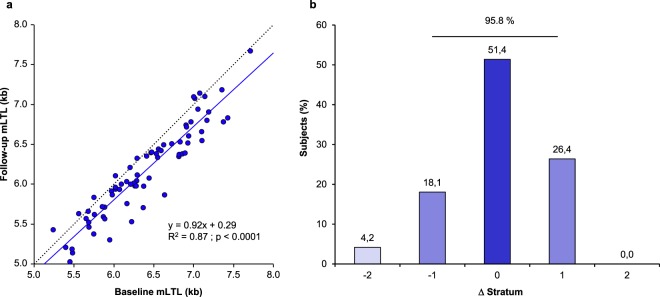
Fixed mLTL ranking over time in the ADELAHYDE cohort. (**a**) Correlation between baseline mLTL and follow-up mLTL. The dashed line represents the identity line. The continuous line represents the linear regression of the data. R^2^ (based on Pearson correlation) is equal to 0.87. (**b**) Distribution of subjects exhibiting a change in stratum rank (Δ) between baseline and follow-up examinations (population subdivided in 7 mLTL strata in each examination). A negative sign denotes a downward shift in ranking while a positive sign indicates an upward shift in ranking. mLTL = mean leukocyte telomere length.

Linear regression was used to assess the association between mLTL at baseline and follow-up. mLTL showed robust tracking, such that individuals with a relatively longer (or shorter) mLTL at baseline also showed a relatively longer (or shorter) mLTL at follow-up (Fig. 1a). The Pearson correlation was r = 0.93. To further examine tracking in the context of the ranking of an individual’s mLTL among his/her peers, individuals were subdivided into seven strata according to their mLTL at baseline and follow-up examinations. Each stratum comprised 10–11 individuals and the change in mLTL rank between baseline and follow-up was examined by stratum change (Fig. 1b). Individuals largely maintained their ranks between baseline and follow-up examinations with 51.4% unchanged, 44.5% showing an increase/decrease of one stratum (26.4% up/18.1% down), and only 4.2% showing a change of 2 or more strata. Thus, 95.8% showed no change in rank or a change of only 1 stratum. The above association was also tested as a continuum in all 72 subjects by performing a non-parametric correlation (Spearman rank analysis). This analysis confirmed the strong relationship between baseline and follow-up ranking (R = 0.94; p < 0.0001).

### LTLD shape

**Figure 2:**
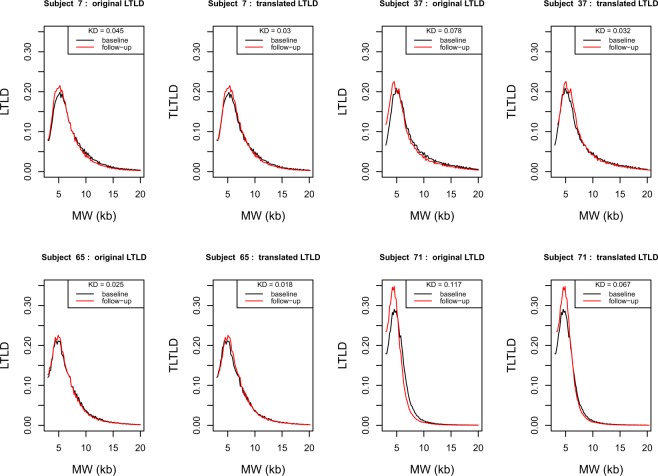
Original LTLD and Translated LTLD (TLTLD) of 4 subjects at baseline and follow-up. LTLD = Leukocyte telomere length distribution; TLTLD = Translated leukocyte telomere length distribution; MW = molecular weight; kb = kilobase; KD = Kolmogorov distance Each. pair of panels shows the two LTLDs of one subject at baseline (in black) and follow-up (in red) and their transformation in TLTLD. The Kolmogorov distances between the two LTLD and the two TLTLD are given. The four subjects are respectively subject 7 (upper left panels), 37 (upper right panels), 65 (lower left panels) and 71 (lower right panels).

To study LTLD shape and its potential reconfiguration over time, the translated LTLDs (TLTLD) at baseline and follow-up were drawn in order to have the same median value in the two distributions. Figure 2 shows examples of this transformation in four subjects (Supplementary Fig. S2 depicts the TLTLD distributions at baseline and follow-up for each of the 72 subjects).

**Figure 3:**
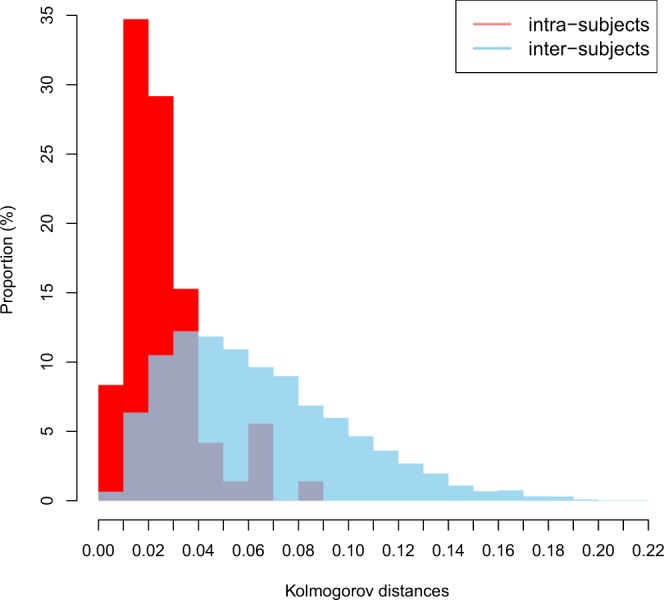
Intra-subject and inter-subject distribution of Kolmogorov distances between TLTLD. Normalised histograms for intra-subject distances (in red, mean = 0.0258) and inter-subject distances (in blue, mean = 0.0639).

Kolmogorov distances between the baseline and follow-up TLTLDs for each of the 72 subjects were computed to illustrate the manner in which the LTLD was reconfigured over the interval of 8 years. The distribution of these 72 distances is shown in red in the normalised histogram in Fig. 3. The 72*71 Kolmogorov distances (each of the 72 subjects was compared with the 71 others) between the TLTLD at baseline for one subject and follow-up TLTD for another subject were also computed. The normalized histogram of these distances is given in blue in Fig. 3. This figure shows that the intra-subject distances were lower than the inter-subject distances: 90% of the intra-subject Kolmogorov distances were lower than 0.05 whereas this was only the case for 40% of the inter-subject distances. The T test confirmed this observation by rejecting the null hypothesis between the two observed means of the intra-subject distances (${\bar{x}}_{n}$= 0.0258) vs. the inter-subject distances (${\bar{y}}_{n}$ = 0.0639) (p < 0.0001). Supplementary Fig. S3 illustrates the TLTLD of one subject taken randomly (number 36) compared to each of the other subjects.

### Relationship between LTLD and mLTL

**Figure 4:**
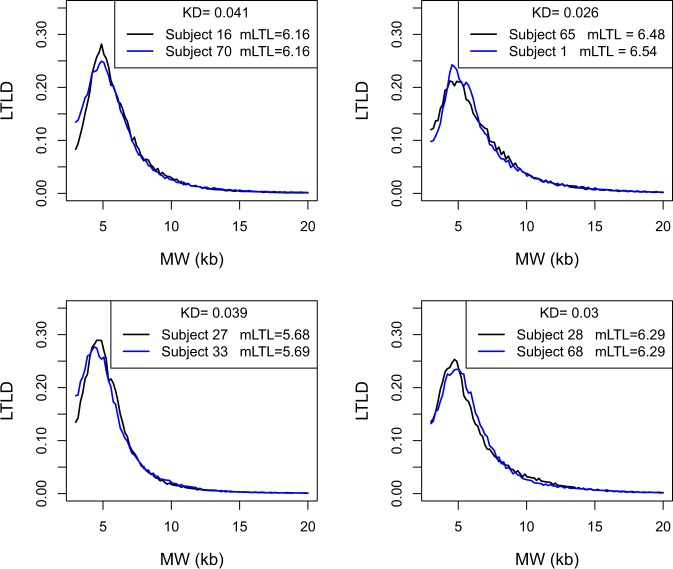
Relationship between LTLD and mLTL. LTLD = Leukocyte telomere length distribution; MW = molecular weight; kb = kilobase; mLTL = mean leukocyte telomere length; KD = Kolmogorov distance. For other abbreviations see legends of Figures 1 and 2. Each panel shows the two LTLD of two subjects with similar mLTL. The Kolmogorov distance between the two LTLD are given.

**Figure 5:**
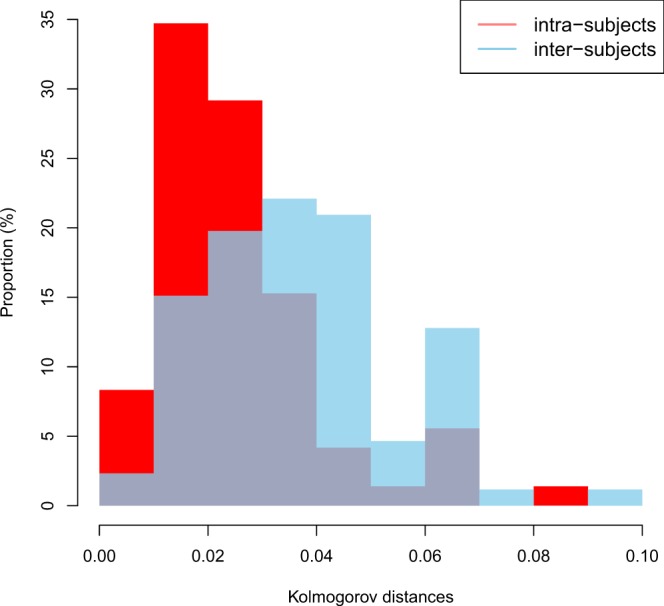
Distributions of Kolmogorov distances (KD) between pairs of individuals with similar mLTL (inter-subject) and between baseline vs. follow-up visits in all individuals (intra-subject). Normalised histograms for intra-subject distances (in red, mean = 0.0258) and inter-subject (with similar mLTL) distances (in blue, mean = 0.0374). Intra-subject KDs measure the differences in TLTLD between baseline and follow-up of the 72 subjects. Inter-subject KDs measure the differences in LTLD of 86 pairs of subjects with similar mLTL (less than 40 bp difference). For abbreviations see legends of Figures 1 and 2.

Figure 4 depicts four pairs of subjects. Each of these pairs had roughly the same values of mLTL at baseline: 6.16 for subjects 16 and 70; approximately 6.51 for subjects 1 and 65; approximately 5.68 for subjects 27 and 33; and 6.29 for subjects 28 and 68. Whereas the mLTL were very similar, the distributions differed considerably as expressed by the Kolmogorov distances. To generalize and confirm these observations, the KDs between 86 pairs of subjects with relatively similar mLTL (less than 40 bp difference in mLTL) were compared with the 72 intra-subject KDs. The mean intra-subject difference in mLTL between baseline and follow-up was 227 bp (more than 5 times higher than the inter-subject differences). Nevertheless, intra-subject KD was statistically lower than the KD between subjects with similar mLTL (0.026 vs. 0.037; p < 0.0001; Fig. 5).

## Discussion

The central finding of this study is that individuals, even those with similar mLTL, display different LTLD shapes. In addition, these shapes are stable over time, therefore the LTLD shape may be characteristic of each individual.

TL plays an important role in morbidity and mortality in humans. Comparatively short mLTL is associated with atherosclerosis^25^, dementia^26^, type 2 diabetes mellitus^27^ and cardiovascular mortality^12^. However, the vast majority of these associations are based on mLTL, which reflects all of the different TL in the chromosomal ends and cells in a given DNA sample. Associations of LTLD with aging-related diseases and longevity have not been examined comprehensively. Some studies have shown that the shortest telomeres, measured using universal STELA, may be associated with senescence or disease whereas mLTL is not^28–30^. One study using TRF to calculate the percentage of short/medium/long telomeres has shown that the distribution can differ between groups with relatively similar mLTL^31^. In contrast, the present study analysed the entire LTL distribution as a continuum. As a result, we were able to describe differences in LTLD shapes among individuals even when their mLTLs were similar.

We have previously shown that mLTL ranking among individuals changes little during adult life^32^ and that during this period, risk factors and diseases have comparatively less effect on mLTL attrition than variations already in place prior to adulthood^33,34^. This indicates that mLTL is primarily determined early in life, mainly by mLTL at birth and its dynamics during the first two decades of life, in order to accommodate hematopoietic stem cell pool expansion^35–37^. However, mLTL attrition rates during adult life do not capture potential reconfiguration in LTLD. Two studies using SB have shown that the distribution between short/medium/long telomeres can change in cultured human cells under specific temperature or radiation conditions^38,39^. In principle, LTLD changes may occur without major impact on mLTL. The contribution of this study is to show a strong stability over time not only in the ranking according to mLTL, but also of the shape of LTLD. Since LTLD could be linked to patterns of stem cell growth^36^, it could hence play a determinant role in disease development.

The main limitation of this study is that the follow-up duration was only 8 years and therefore we cannot conclude on variations occurring over a longer period. Another limitation is related to the selected profile of the cohort and therefore these analyses should be reproduced in larger unselected cohorts. Third, the Southern blot method lacks sensitivity to detect very short telomeres (below 3 kb) which are therefore excluded from the observed LTLD.

In light of the present findings, we propose that in future clinical studies, LTLD signature could capture new associations between TL dynamics and clinical parameters or disease markers. LTLD signature could become a new parameter to describe patients, enabling a better clustering of and/or discrimination between these patients, thus enabling better insight into the TL-disease connection.

## Methods

### Subjects

Leukocyte DNA was extracted from blood samples donated by participants in the ADELAHYDE (Analyse des DEterminants génétiques et environnementaux de la Leucoaraïose dans une population à sujets Agés HYpertendus présentant des troubles cognitifs DEbutants) study. The aim of the study was to assess the role of vascular and genetic parameters in the development of cognitive decline in old hypertensive subjects^40^. Participants were men and women aged over 60 years at baseline examination with a history of hypertension and subjective cognitive complaint. A total of 378 hypertensive men and women were recruited between September 2003 and August 2005 (baseline examination); of these, 131 had a follow-up examinations 8 years later between 2011 and 2013^41^. Among these patients (50% women), 72 had available DNA in sufficient quality and quantity to perform LTL measurements from both baseline and follow-up examinations. The results of these 72 subjects are included in the present analysis. Mean age at baseline examination was 68 ± 5 (SD) years (age range at 60–81 years). The study was approved by the local Research Ethics Committee (Comité de Protection des Personnes de Nancy, France) and all research was performed in accordance with relevant guidelines and regulations. All participants signed an informed consent form.

### Leukocyte telomere length (LTL) measurements

White blood cell DNA was extracted from whole blood after red cell osmotic lysis by a salting out method as described previously^42^. All DNA samples were verified for integrity by resolving 100 ng of each sample on a 1% (wt/vol) agarose gel. LTL was measured by Southern blot of the TRF, as described previously^11^. Briefly, DNA samples were digested (37 °C) overnight with the restriction enzymes Hinf I and Rsa I (Roche Diagnostics GmbH, Germany). Digested DNA samples and DNA ladders were resolved on 0.5% (wt/vol) agarose gels. After 23 h, the DNA was depurinated, denatured, neutralised and transferred onto a positively charged nylon membrane (Roche) using a vacuum blotter (Biorad, Hercules, CA). Membranes were hybridised at 42 °C with a DIG-labelled telomeric probe after which the probe was detected by the DIG luminescent detection procedure (Roche) and exposed on CCD camera (Las 4000, Fuji). Optical density values (OD) versus DNA migration distances obtained from raw data were converted to OD/MW versus MW using a 4^th^ degree polynomial function transformation of DNA migration distance (mm) in MW (kb) owing to DNA ladders. Mean TRF was calculated and the TRF distribution observed in the 3 kb–20 kb range.

Baseline and follow-up samples from each subject were run in adjacent lanes (Supplementary Fig. S1). Measurements were performed in duplicate on separate membranes with an inter-assay variation coefficient of 1.2% on mLTL.

### Leukocyte Telomere Length Distribution (LTLD) computations

The proportion of leukocyte telomeres with weight MW was calculated for each MW value between the 3 kb–20 kb range for a given DNA sample (Fig. S1); the LTL distribution (LTLD) was obtained as the function:

$$LTLD(MW)=\frac{OD/MW}{T},\,{\rm{where}}\,T={\sum }^{}OD/MW$$

where for each MW value, OD/MW is proportional to the number of leukocyte telomeres with weight MW in the DNA sample and LTLD (MW) ranges between 0 and 1.

#### Properties of the LTLD function

This approach provides different information than the OD, since LTLD (MW) is a relative value taking into account the entire range of the TRF between 3 kb–20 kb, contrary to the OD value which is dependent on measurement conditions and starting material. For example, if LTLD(MW1) > LTLD(MW2) for two different values of MW1 and MW2, this indicates that there are more telomeres with weight MW1 than telomeres with weight MW2 in the DNA sample. This information is not provided by the corresponding values of OD1 and OD2. Indeed, OD is only proportional to the number of kb (number of telomeres*length of telomeres).

LTLD therefore represents a probability distribution over the values of MW in the 3 kb–20 kb range of the DNA sample. The mean of this LTLD is obtained by the formula:

$$Mean(TLD)=\sum _{MW}MW\frac{OD/MW}{T}=\frac{{\sum }_{MW}OD}{{\sum }_{MW}OD/MW}$$

and corresponds to the mean TRF value, typically calculated and generally used in the analysis (mLTL). The variance is obtained by the following formula:

$$Var(TLD)=\frac{{\sum }_{MW}OD\ast MW}{{\sum }_{MW}OD/MW}-{(\frac{{\sum }_{MW}OD}{{\sum }_{MW}OD/MW})}^{2}$$

The LTLD is a probability distribution, whose cumulative distribution function (CDF) is defined as the function:

$$CDF(MW)=\sum _{MW^{\prime} \le MW}LTLD(M{W}^{\text{'}})$$

For a given MW, CDF(MW) is the proportion of values that are less than MW in the DNA sample. For any value p in [0,1], the p-quantile of the LTLD is

$${q}_{p}=inf\{MW,\,{\rm{such}}\,{\rm{that}}\,CDF(MW)\ge p\}$$

and a proportion p of leukocyte telomeres in the sample is smaller than q_p_. In particular, q_1/2_ is the median of the leukocyte telomere lengths of the current DNA sample.

#### Translation for all DNA samples

In order to study the shape of the distribution (disregarding the central parameters) and allow comparisons between DNA samples, a translated telomere length distribution (TLTLD) was defined as follows:

$$TLTLD(MW)=LTLD(MW+{q}_{1/2}-5.5).$$

This is a probability distribution with a median equal to 5.5 kb. This translation was applied to each DNA sample.

TLTLD has the same shape as the original distribution (LTLD) and the choice of 5.5 kb does not change the result or the analysis. Indeed, this value only translates all of the TLTLD by a same amount of kb, which could have been 10 kb or 20 kb. The 5.5 kb value was chosen given its proximity to the median of most measured LTLD. LTLD and TLTLD are graphically represented by their density

#### The Kolmogorov distance

To allow comparison of the shape between DNA samples, the Kolmogorov distance^43^ between the probability TLTLD measurements for two different DNA samples was chosen, and defined as:

$$Kol(TLTL{D}_{1},TLTL{D}_{2})=ma{x}_{MW}(CD{F}_{1}(MW+{q}_{1/2}-5.5)-CD{F}_{2}(MW+{q}_{1/2}-5.5))$$

Note that the value of Kol(TLTLD_1_, TLTLD_2_) is independent of the choice of 5.5 kb of the translation.

### Statistical analysis

Descriptive values are expressed as mean ± standard deviation (SD) and percentages. mLTL attrition was calculated from the difference between mLTL at baseline and mLTL at follow-up, divided by the duration of the follow-up.

The relationship between mLTL at baseline and mLTL at follow-up was determined using Pearson’s correlation coefficients. Paired T tests were performed to compare parameters between baseline and follow-up visits. T tests were performed to compare intra-subject Kolmogorov distances versus inter-subject Kolmogorov distances. A p-value < 0.05 was regarded as statistically significant. Statistical analyses were carried out using the NCSS 9 statistical software package (NCSS, Kaysville, UT) and R statistical software.

## Electronic supplementary material

Supplementary information

## Data Availability

The R script and datasets generated during and/or analysed during the current study are available from the corresponding author on reasonable request.
