# Supplementary material for: The individual’s signature of telomere length distribution
Source: Sci Rep. 2019 Jan 24;9:685. doi: 10.1038/s41598-018-36756-8 (PMC6345926; doi:10.1038/s41598-018-36756-8)
Supplement: Supplementary file 1 — Supplementary information [file 41598_2018_36756_MOESM1_ESM.pdf]

Supplementary information

The individual's signature of telomere length distribution

Simon TOUPANCE[1,2,3], Denis VILLEMONAIS[4,5], Daphné GERMAIN[4], Anne GEGOUT-PETIT[5], Eliane ALBUISSON[5,6,7] and Athanase BENETOS[1,2 *].

1. Université de Lorraine, Inserm, DCAC, F-54000 Nancy, France ;

2. Université de Lorraine, CHRU-Nancy, Pôle "Maladies du Vieillissement, Gérontologie et Soins Palliatifs", F-54000, France ;

3. Nancyclotep-GIE, F-54000 Nancy, France ;

4. Université de Lorraine, Ecole des Mines, F-54000 Nancy, France ;

5. Université de Lorraine, CNRS, Inria, IECL, F-54000 Nancy, France ;

6. Université de Lorraine, CHRU de Nancy, BIOBASE, Pôle S2R, F-54000, France ;

7. Université de Lorraine, InSciDenSe, F-54000 Nancy, France.

Supplementary Figure S1: Illustrative membrane of Southern blots of the terminal restriction fragments (TRFs) from the ADELAHYDE study.

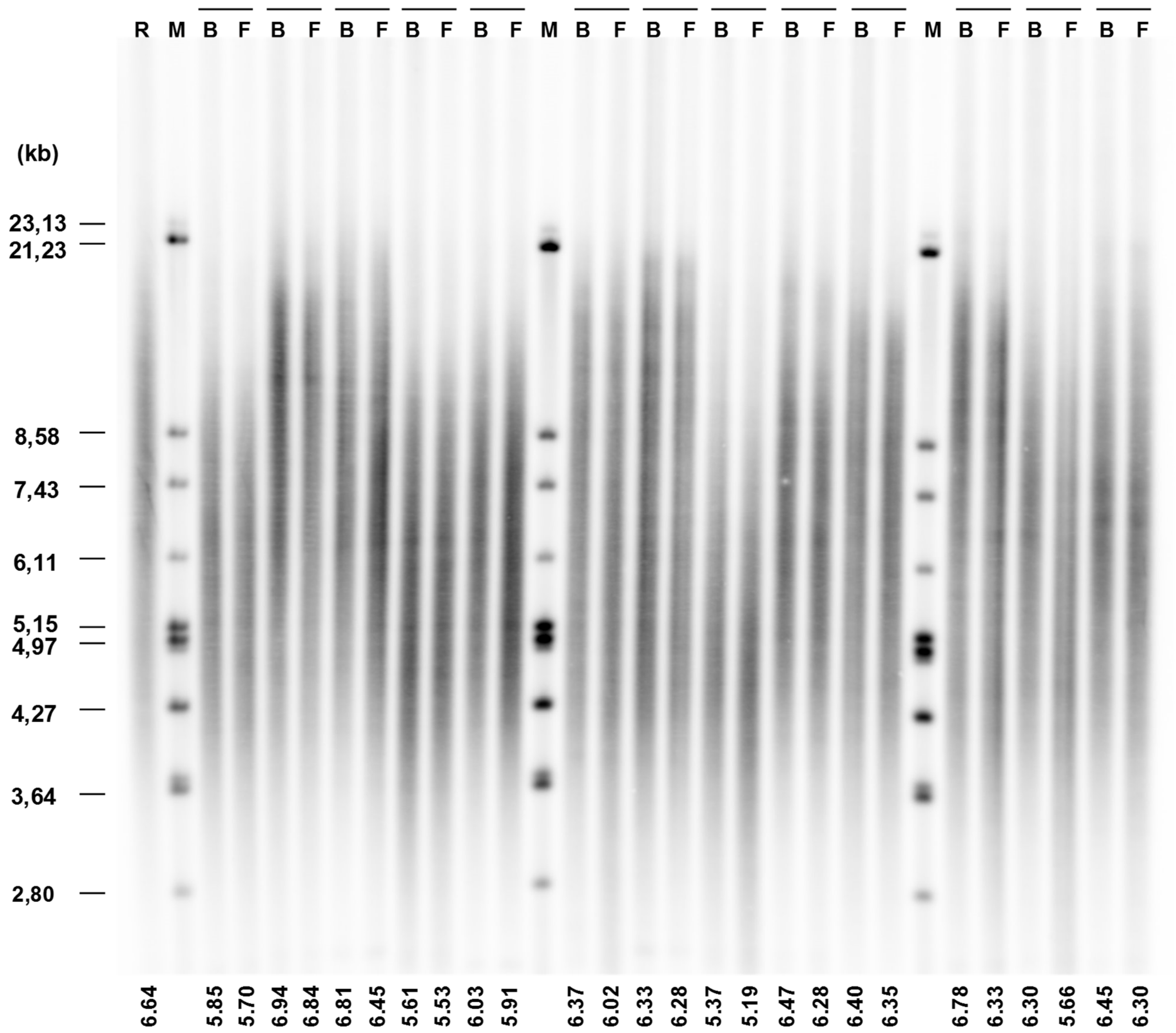

A sample of known TRF length served as an internal reference (R). Three molecular weight (M) ladders were resolved at regular intervals across the gel, with the one closest to a given sample used for the computation of the distance to molecular weight for that sample. Mean baseline (B) and follow-up (F) TRFs are shown (in kb) at the bottom of the lanes.

Supplementary Figure S2: Translated LTL distributions at baseline and follow-up for each of the 72 subjects.

TLTLD = Translated leukocyte telomere length distribution; MW = molecular weight; kb = kilobase; KD = Kolmogorov distance.

Each panel shows the two TLTLDs of a subject at baseline (in black) and follow-up (in red). The Kolmogorov distances between the two TLTLD are given. The Kolmogorov distance analysis shows a very similar LTL distribution in each subject over a period of 8 years (See figure 3).

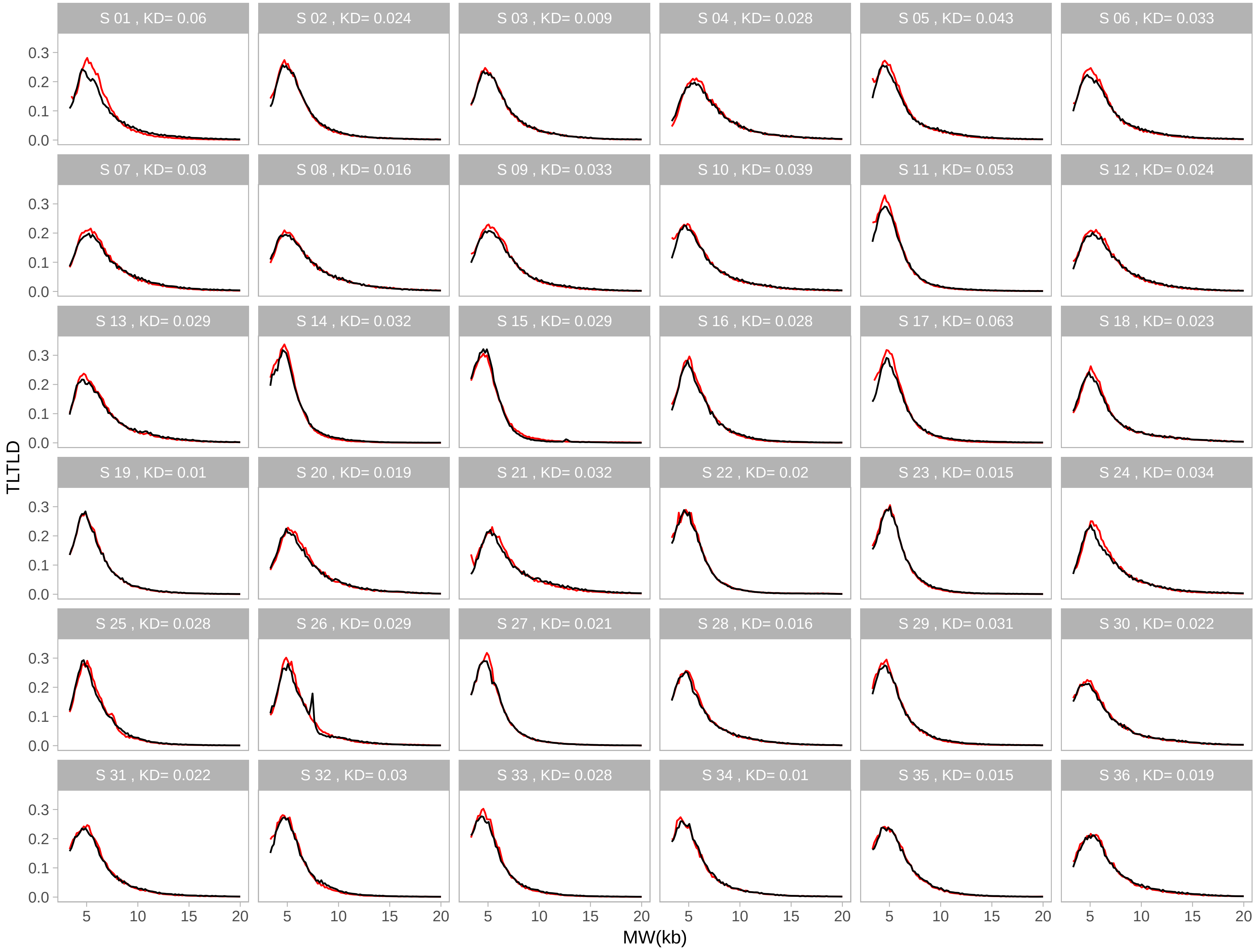

S 01 , KD= 0.06
S 02 , KD= 0.024
S 03 , KD= 0.009
S 04 , KD= 0.028
S 05 , KD= 0.043
S 06 , KD= 0.033
S 07 , KD= 0.03
S 08 , KD= 0.016
S 09 , KD= 0.033
S 10 , KD= 0.039
S 11 , KD= 0.053
S 12 , KD= 0.024
S 13 , KD= 0.029
S 14 , KD= 0.032
S 15 , KD= 0.029
S 16 , KD= 0.028
S 17 , KD= 0.063
S 18 , KD= 0.023
S 19 , KD= 0.01
S 20 , KD= 0.019
S 21 , KD= 0.032
S 22 , KD= 0.02
S 23 , KD= 0.015
S 24 , KD= 0.034
S 25 , KD= 0.028
S 26 , KD= 0.029
S 27 , KD= 0.021
S 28 , KD= 0.016
S 29 , KD= 0.031
S 30 , KD= 0.022
S 31 , KD= 0.022
S 32 , KD= 0.03
S 33 , KD= 0.028
S 34 , KD= 0.01
S 35 , KD= 0.015
S 36 , KD= 0.019
TLTLD
0.0
0.1
0.2
0.3
MW(kb)
5
10
15
20

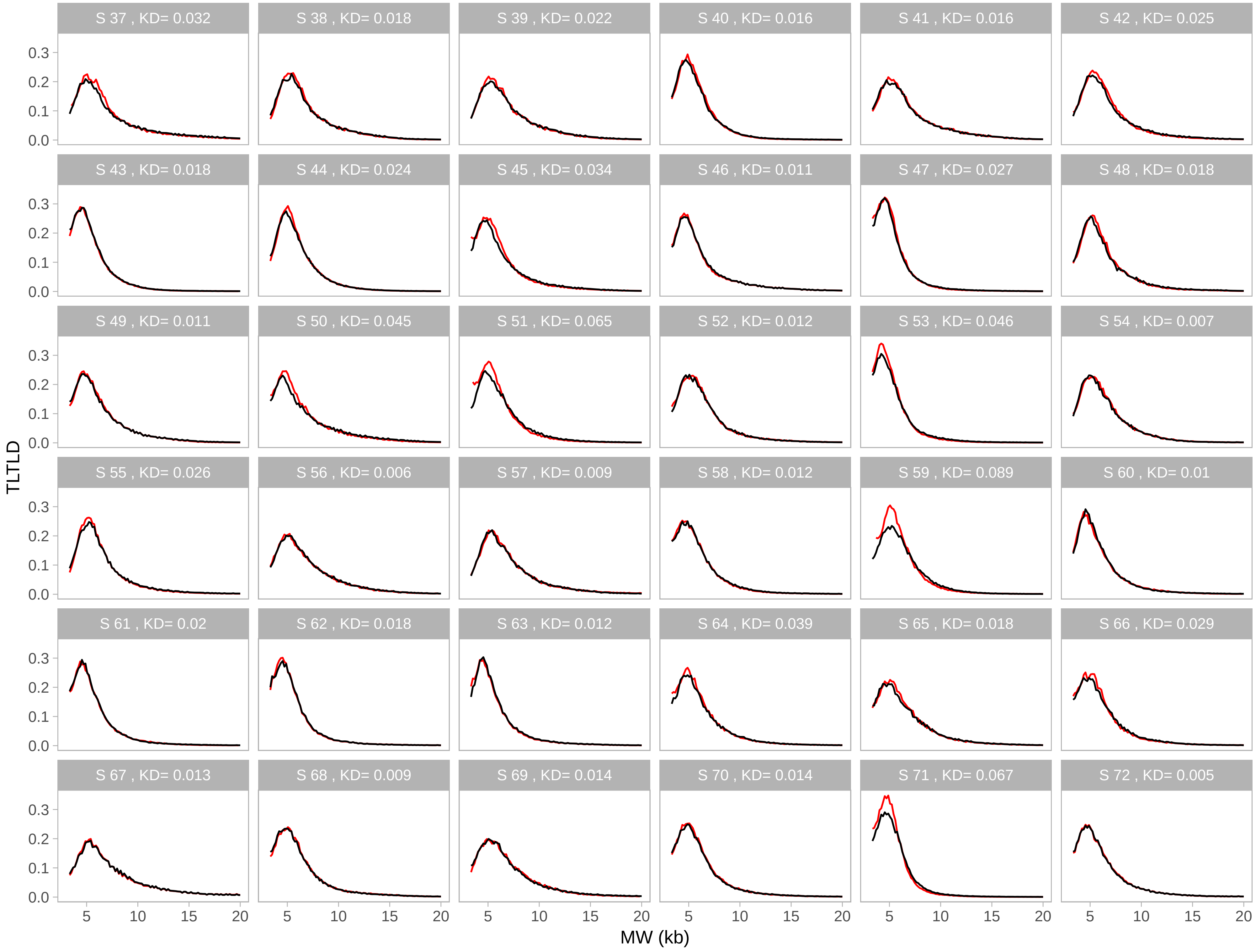
S 37 , KD= 0.032
S 38 , KD= 0.018
S 39 , KD= 0.022
S 40 , KD= 0.016
S 41 , KD= 0.016
S 42 , KD= 0.025
S 43 , KD= 0.018
S 44 , KD= 0.024
S 45 , KD= 0.034
S 46 , KD= 0.011
S 47 , KD= 0.027
S 48 , KD= 0.018
S 49 , KD= 0.011
S 50 , KD= 0.045
S 51 , KD= 0.065
S 52 , KD= 0.012
S 53 , KD= 0.046
S 54 , KD= 0.007
S 55 , KD= 0.026
S 56 , KD= 0.006
S 57 , KD= 0.009
S 58 , KD= 0.012
S 59 , KD= 0.089
S 60 , KD= 0.01
S 61 , KD= 0.02
S 62 , KD= 0.018
S 63 , KD= 0.012
S 64 , KD= 0.039
S 65 , KD= 0.018
S 66 , KD= 0.029
S 67 , KD= 0.013
S 68 , KD= 0.009
S 69 , KD= 0.014
S 70 , KD= 0.014
S 71 , KD= 0.067
S 72 , KD= 0.005
TLTLD
0.0
0.1
0.2
0.3
5
10
15
20
MW (kb)

Supplementary Figure S3: Comparison of translated LTL distributions between subject 36 and each of the remaining study subjects.

TLTLD = Translated leukocyte telomere length distribution; MW = molecular weight; kb = kilobase; KD = Kolmogorov distance.

The first upper left image indicates subject 36 at baseline (black line) vs. follow-up (blue line). All other images depict subject 36 at baseline (black line) vs. the 71 other subjects at follow up (blue lines). The Kolmogorov distances between the two TLTLD are given.

The KD analysis shows higher inter-individual than intra-individual differences (see figure 3).

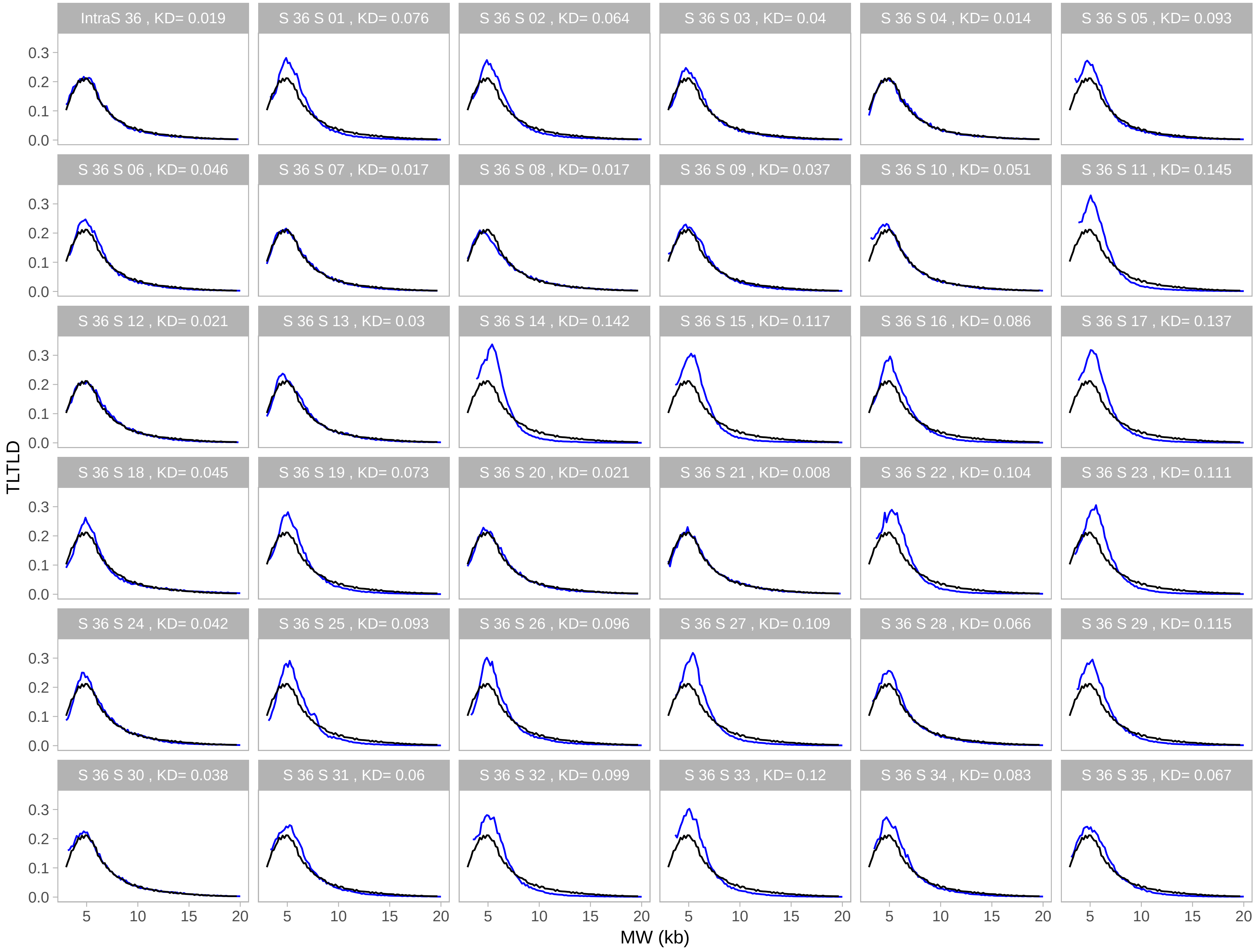
IntraS 36 , KD= 0.019
S 36 S 01 , KD= 0.076
S 36 S 02 , KD= 0.064
S 36 S 03 , KD= 0.04
S 36 S 04 , KD= 0.014
S 36 S 05 , KD= 0.093
S 36 S 06 , KD= 0.046
S 36 S 07 , KD= 0.017
S 36 S 08 , KD= 0.017
S 36 S 09 , KD= 0.037
S 36 S 10 , KD= 0.051
S 36 S 11 , KD= 0.145
S 36 S 12 , KD= 0.021
S 36 S 13 , KD= 0.03
S 36 S 14 , KD= 0.142
S 36 S 15 , KD= 0.117
S 36 S 16 , KD= 0.086
S 36 S 17 , KD= 0.137
S 36 S 18 , KD= 0.045
S 36 S 19 , KD= 0.073
S 36 S 20 , KD= 0.021
S 36 S 21 , KD= 0.008
S 36 S 22 , KD= 0.104
S 36 S 23 , KD= 0.111
S 36 S 24 , KD= 0.042
S 36 S 25 , KD= 0.093
S 36 S 26 , KD= 0.096
S 36 S 27 , KD= 0.109
S 36 S 28 , KD= 0.066
S 36 S 29 , KD= 0.115
S 36 S 30 , KD= 0.038
S 36 S 31 , KD= 0.06
S 36 S 32 , KD= 0.099
S 36 S 33 , KD= 0.12
S 36 S 34 , KD= 0.083
S 36 S 35 , KD= 0.067
TLTLD
0.0
0.1
0.2
0.3
MW (kb)
5
10
15
20

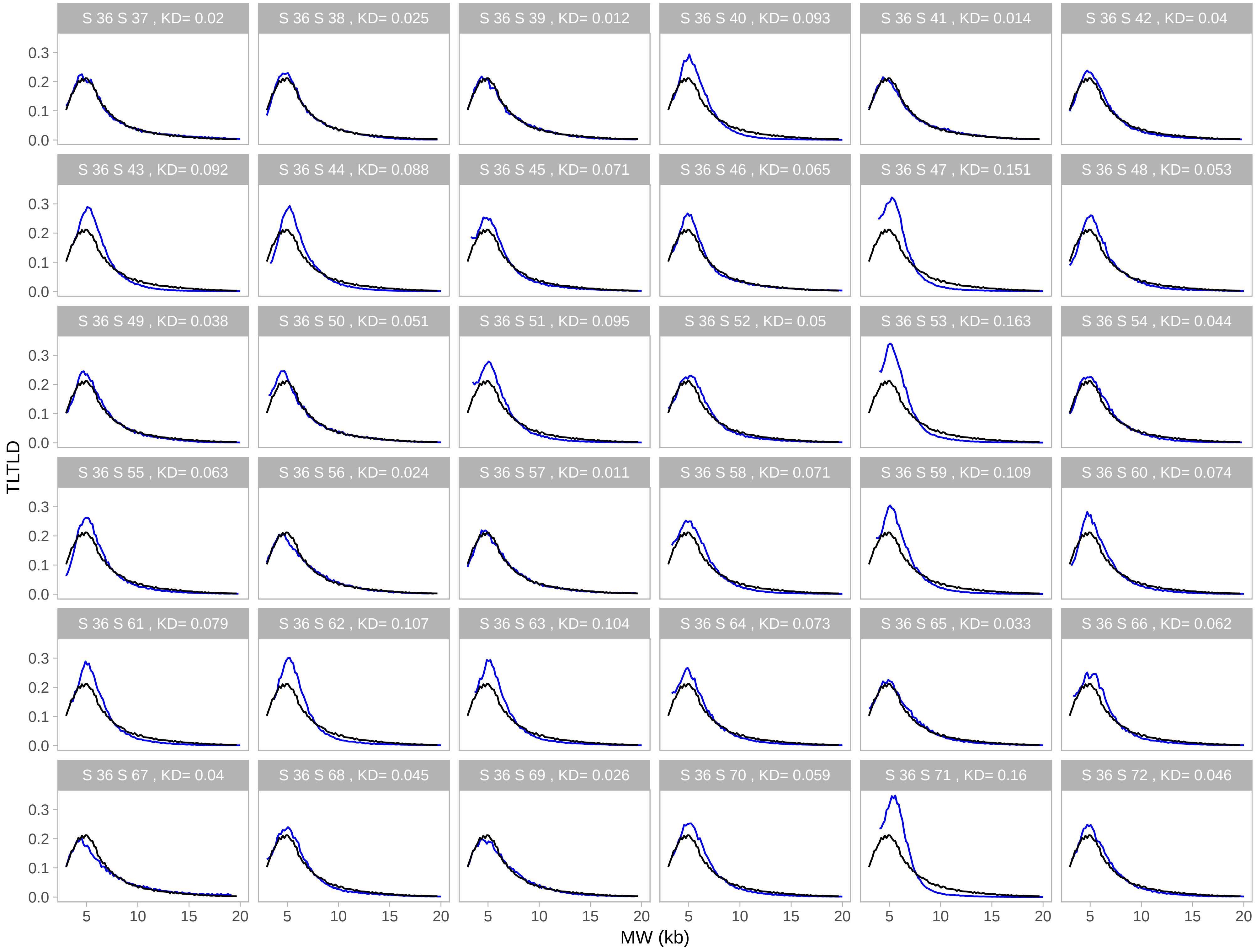

S 36 S 37 , KD= 0.02
S 36 S 38 , KD= 0.025
S 36 S 39 , KD= 0.012
S 36 S 40 , KD= 0.093
S 36 S 41 , KD= 0.014
S 36 S 42 , KD= 0.04
S 36 S 43 , KD= 0.092
S 36 S 44 , KD= 0.088
S 36 S 45 , KD= 0.071
S 36 S 46 , KD= 0.065
S 36 S 47 , KD= 0.151
S 36 S 48 , KD= 0.053
S 36 S 49 , KD= 0.038
S 36 S 50 , KD= 0.051
S 36 S 51 , KD= 0.095
S 36 S 52 , KD= 0.05
S 36 S 53 , KD= 0.163
S 36 S 54 , KD= 0.044
S 36 S 55 , KD= 0.063
S 36 S 56 , KD= 0.024
S 36 S 57 , KD= 0.011
S 36 S 58 , KD= 0.071
S 36 S 59 , KD= 0.109
S 36 S 60 , KD= 0.074
S 36 S 61 , KD= 0.079
S 36 S 62 , KD= 0.107
S 36 S 63 , KD= 0.104
S 36 S 64 , KD= 0.073
S 36 S 65 , KD= 0.033
S 36 S 66 , KD= 0.062
S 36 S 67 , KD= 0.04
S 36 S 68 , KD= 0.045
S 36 S 69 , KD= 0.026
S 36 S 70 , KD= 0.059
S 36 S 71 , KD= 0.16
S 36 S 72 , KD= 0.046
TLTLD
0.0
0.1
0.2
0.3
MW (kb)
5
10
15
20
